# A genetic study of a Brazilian cohort of patients with X-linked hypophosphatemia reveals no correlation between genotype and phenotype

**DOI:** 10.3389/fped.2023.1215952

**Published:** 2023-09-19

**Authors:** Mauro Borghi, Leopoldo Muniz da Silva, Luciana Bispo, Carlos A. Longui

**Affiliations:** ^1^ School of Medical Sciences Santa Casa SP and Pediatric Endocrinology Unit, Irmandade da Santa Casa de Misericórdia de São Paulo, São Paulo, Brazil; ^2^ Hospital São Luiz—Rede D´Or—CMA, Departament of Anesthesiology, São Paulo, Brazil; ^3^ Laboratório Mendelics, Department of Genetic, São Paulo, Brazil

**Keywords:** genotype, genotype–phenotype correlation, phosphate, variants, X-linked hypophosphatemic rickets, phenotype

## Abstract

**Aim:**

X-linked hypophosphatemia (XLH) is the most common inherited form of rickets, and it is caused by pathogenic inactivating variants of the phosphate-regulating endopeptidase homolog X-linked (*PHEX*) gene. The main purpose of this study is to identify the presence of a genotype–phenotype correlation in a cohort of XLH patients.

**Methods:**

This is a retrospective study including patients diagnosed with hypophosphatemic rickets, confirmed by clinical, radiological, and laboratory findings. Medical records were reviewed for phenotypic analyses. Genomic DNA was extracted from the peripheral blood lymphocytes, and *PHEX* sequencing was performed by exomic NGS sequencing. The Wilcoxon rank-sum test and the two-tailed Fisher's exact test were employed for the statistical analyses of this study.

**Results:**

A total of 41 patients were included in this study, and 63.41% (26/41) of the patients were female. The mutation analyses identified 29.27% missense variants and 29.72% nonsense variants, most of them were considered deleterious (66.41%). Six novel deleterious variants in the *PHEX* gene were detected in seven patients. The median concentrations of pretreatment serum calcium, phosphorus, and parathyroid hormone (PTH) were not significantly different among patients with different genotypes. An orthopedic surgery due to bone deformity was required in 57.69%.

**Conclusions:**

Our analysis did not identify any specific genotype as a predictor. No significant genotype–phenotype correlation was found, suggesting that the recognition of subjacent pathogenic mutation in the *PHEX* gene may have limited prognostic value. Despite this finding, genetic testing may be useful for identifying affected individuals early and providing appropriate treatment.

## 1. Introduction

X-linked hypophosphatemia (XLH) is the most common inherited form of hypophosphatemic rickets, with an incidence of 1:20,000 individuals (1–4). XLH is caused by inactivating pathogenic variants in the phosphate-regulating endopeptidase homolog X-linked (*PHEX*) gene, which is located on chromosome Xp22.1 and is encoded by 22 exons (5). Different gene defects including nonsense, frameshift insertion/deletions, or splicing variants result in a truncating form of the PHEX protein, which is a very likely deleterious form, whereas a non-truncating form of the protein arises from the missense gene variant (6, 7).

Although XLH rickets is inherited in an X-linked dominant trait, the severity of its manifestations varies and will depend on the profile of the studied population (7). The clinical history of XLH is heterogeneous, as the severity of growth failure and skeletal deformity varies between affected individuals (7). The genotype–phenotype correlations are relatively well-established in several other X-linked genetic diseases, with the truncating variants related to a more severe phenotype compared with the non-truncating variants (8, 9). Some authors have also reported a genotype–phenotype correlation in patients with XLH (7–9). For instance, orthopedic surgery was significantly more often required in patients with truncating variants (9). Song et al. (10) revealed that variants in the C-terminal region of the *PHEX* gene were associated with a more severe bone phenotype.

Considerable inconsistency exists regarding the correlation of the truncating and non-truncating variant groups, and their disease phenotype as previously reported (7–10). Therefore, the aim of this study was to evaluate the presence of genotype–phenotype correlation in a cohort of XLH patients under long-term follow-up.

## 2. Materials and methods

This retrospective observational cohort study comprised 41 XLH-diagnosed patients followed up from the period of 1971 to 2018 in the Bone Metabolism Clinic of Irmandade da Santa Casa de Misericordia of São Paulo, Brazil. Informed consent was obtained from the patients or parents. This study protocol was approved by the Institutional Review Board (protocol no. #02067418.5.0000.5479, assent CEP 3.225.914), and the study was compliant with the resolution 466/2012 of the Brazilian National Health Council.

### 2.1. Clinical and skeletal features

The medical records were retrospectively reviewed to obtain demographic information as well as clinical and biochemical manifestations at the time of diagnosis. XLH diagnosis was based on clinical, radiological, and biochemical findings. Clinical features and *PHEX* gene variants, as well as the clinical and surgical outcome of the patient, were recorded over the time. Anthropometric measurements and biochemical parameters were obtained at diagnosis and prior to any treatment. The data evaluated for the genotype–phenotype correlation were gender, height, body mass index (BMI), and the serum concentrations of calcium, phosphorus, parathyroid hormone (PTH), and alkaline phosphatase. Stature and weight were measured by trained physicians, according to standardized procedures. Children younger than 2 years of age were measured in the lying down position; children older than 2 years were measured in the standing position by employing a wall stadiometer (Harpenden standard). Anthropometric indices were expressed as a standard deviation score (SDS, *Z*-score). Data from the Centers for Disease Control and Prevention (CDC, release 2000) were adopted as a reference population (11).

To determine the radiological features, the interpatellar distance was measured between the half-point of the patella of each limb, with the patient standing in the anterior position. The intercondylar distance was measured between the anterior and posterior condylar depressions (Supplementary Figure S1). Distances were measured using a tape or rigid ruler graduated in millimeters, and were confirmed by two observers. Orthopedic surgery due to bone deformity was considered as a dichotomous variable, and the type of procedure was not studied.

### 2.2. Biochemical analysis

An evaluation of biochemical parameters in the blood was performed, utilizing the following method for each parameter: calcium (mg/dL; colorimetric assay); phosphorus (mg/dL; kinetic colorimetric assay); alkaline phosphatase (UI/L; photometric assay with ultraviolet range, Cobas C501, Roche Diagnosis, Basel, Switzerland); and PTH (pg/mL; electro-chemiluminometric assay; Cobas E411 Roche Diagnosis, Basel, Switzerland). All measurements were performed in the basal condition at the time of diagnosis, before any medication intervention.

### 2.3. *PHEX* sequencing and variant analysis

Genomic DNA was extracted from the peripheral blood lymphocytes using the G-DEXTM, II Genomic DNA Extraction Kit (Intron, Seongnam, Korea), according to the manufacturer's protocol. After DNA extraction, the regions of interest were captured with probes customized with an Illumina Nextera Rapid Capture kit (Illumina, Inc., San Diego, CA, USA). The sequencing process was performed using Illumina HiSeq 4000. The resultant sequence reads were aligned to the reference human genome (UCSC Genome Browser GrCh37/hg19) using the BWA (Burrows–Wheeler Aligner) software. Genotyping was conducted by utilizing the genome analysis toolkit (GATK). Enrichment and analysis focused on coding sequences, flanking intronic regions (±20 bp), and other specific genomic regions previously identified as containing causative variants. Furthermore, annotation, filtering, and variant prioritization were performed using Mendelics proprietary software (Abracadabra)®.

Only the sequencing of the *PHEX* gene was performed in some patients, while other patients underwent a multigene panel analysis comprising 13 specifically chosen genes. This method was preferred since these conditions are potential differential diagnoses for X-linked hypophosphatemic rickets. The following genes were included: *ALPL* (OMIM* 171760), *CLCN5* (OMIM* 300008), *CYP27B1* (OMIM* 609506), *CYP2R1* (OMIM* 608713), *DMP1* (OMIM* 600980), *ENPP1* (OMIM* 173335), *FAH* (OMIM* 613871), *FGF23* (OMIM* 605380), *KL* (OMIM* 604824), *PHEX* (OMIM* 300550), *SLC34A1* (OMIM* 182309), *SLC34A3* (OMIM* 609826), and *VDR* (OMIM* 601769).

Exonic deletions and duplications (copy number variations—CNVs) were detected using ExomeDepth, an R package that calculates copy number by comparing the read depth of each target with the average read depth of the corresponding target in samples genotyped from the same sequenced library.

The variants were classified using machine learning algorithms developed by Mendelics Genomic Analysis (Sao Paulo, Brazil), and all variants were assessed by medical geneticists, following the consensus of the American College of Medical Genetics and Genomics and the Association for Molecular Pathology (12, 13). Variants were classified as pathogenic, likely pathogenic, or variants of unknown significance (VUS) (13).

Several tools were employed for variant and gene interpretation in this study, including the utilization of The Genome Aggregation Database versions v2.1.1 and v3.1.2 (14) for allele frequency assessment. For characterizing the nomenclature of sequence variants, we followed the recommendations from the Human Genome Variation Society (HGVS). The ensemble transcript used for the PHEX gene was ENST00000379374. Moreover, specific *in silico* predictors or meta-predictors such as Mutation Taster, SpliceAI Lookup, Human Splicing Finder, and REVEL were taken into consideration (15–17).

To obtain information from medical literature and conduct a comprehensive review, we accessed various databases including ClinVar (18), the Human Gene Mutation Database (HGMD) (19), *PHEX* Locus Specific Database (20), Online Mendelian Inheritance in Man (OMIM), Mastermind (21), and pertinent PubMed articles (15–21). To evaluate the effect of the variant type on the phenotype, we classified the variants into two categories: truncating variants, including nonsense, frameshift, and splice-site alterations; and non-truncating variants, comprising previously reported missense variants (7, 10, 22).

### 2.4. Statistical analysis

Categorical variables were presented as absolute values and percentages, and continuous variables were represented as medians and 25%–75% percentiles. Histograms and the Shapiro–Wilk test were used to assess data distribution. The correlation between continuous variables was assessed using Spearman's correlation coefficient. Analyses of variance were performed using the Kruskal–Wallis test, followed by the Dunn's *post hoc* test in case of *p* < 0.05. Two groups were compared using Mann–Whitney *U* test, as appropriate. The Chi-square test was used to analyze the categorical variables, and the partitioning Chi-square was used when the *p*-value was less than 0.05. The Fisher's exact test was used when one of the expected frequencies was lower than 5. *P*-values of <0.05 were considered statistically significant. Statistical analysis was performed using STATA 16.0 software (Stata Corp, College Station, TX, USA) and R-project version 4.0.2 (R core team, Vienna, Austria). The research data related to this submission have been published in Mendeley Data (https://data.mendeley.com/drafts/b392svv9ns; DOI: 10.17632/b392svv9ns.1). The files associated with this dataset are licensed under an attribution non-commercial 3.0 unported license (CC BY NC 3.0).

## 3. Results

### 3.1. Baseline characteristics and biochemical parameters

A total of 41 patients were treated in a single pediatric unit due to inherited metabolic bone diseases and presented features commonly associated to XLH. The baseline characteristics of this cohort show that 63.4% (26/41) were female. The average age at the XLH diagnosis was 49 months; 72% of the patients were diagnosed within the first 5 years of life, and the majority (57.7%) presented deformities in the lower limbs confirmed by radiological examination at the time of admission. Patients with final height corresponded to 41.5% of the cases (17/41), and 55.5% (15/27) of the patients were reported with a family history of XLH. The pedigrees of the families were shown in Supplementary Figure S2. There were 12 cases that appeared to be sporadic, since no family history of the disease was reported.

Table 1 summarizes the biochemical findings in our cohort. The average serum phosphorus concentration was found to be below the established reference range, with 100% of the patients exhibiting hypophosphatemia. Moreover, 80% of the cases demonstrated serum alkaline phosphatase values exceeding the upper limit of normality. Regarding serum PTH concentrations, 40% of the patients surpassed the upper limit of normality. Elevated concentrations of PTH and alkaline phosphatase were observed in 20% of the cases.

**Table 1:** Biochemical parameters at diagnosis for patients with a *PHEX* gene variant.

Parameters	XLH patients	Reference range
Calcium (mg/dL)	9.6 (9.25–10)	8.6–10.2 (0–14 year)
Phosphorus (mg/dL)	2.6 (2.4–2.9)	4.5–6.5 (0–14 year)
Alkaline phosphatase (UI/L)	750 (413.5–1,350)	42–390 (0–15 year)
PTH (pg/mL)	61.25 (42.17–76.0)	12–68

Values expressed as median (percentile 25%–75%).

### 3.2. Variant analysis of the *PHEX* gene

**Figure 1:**
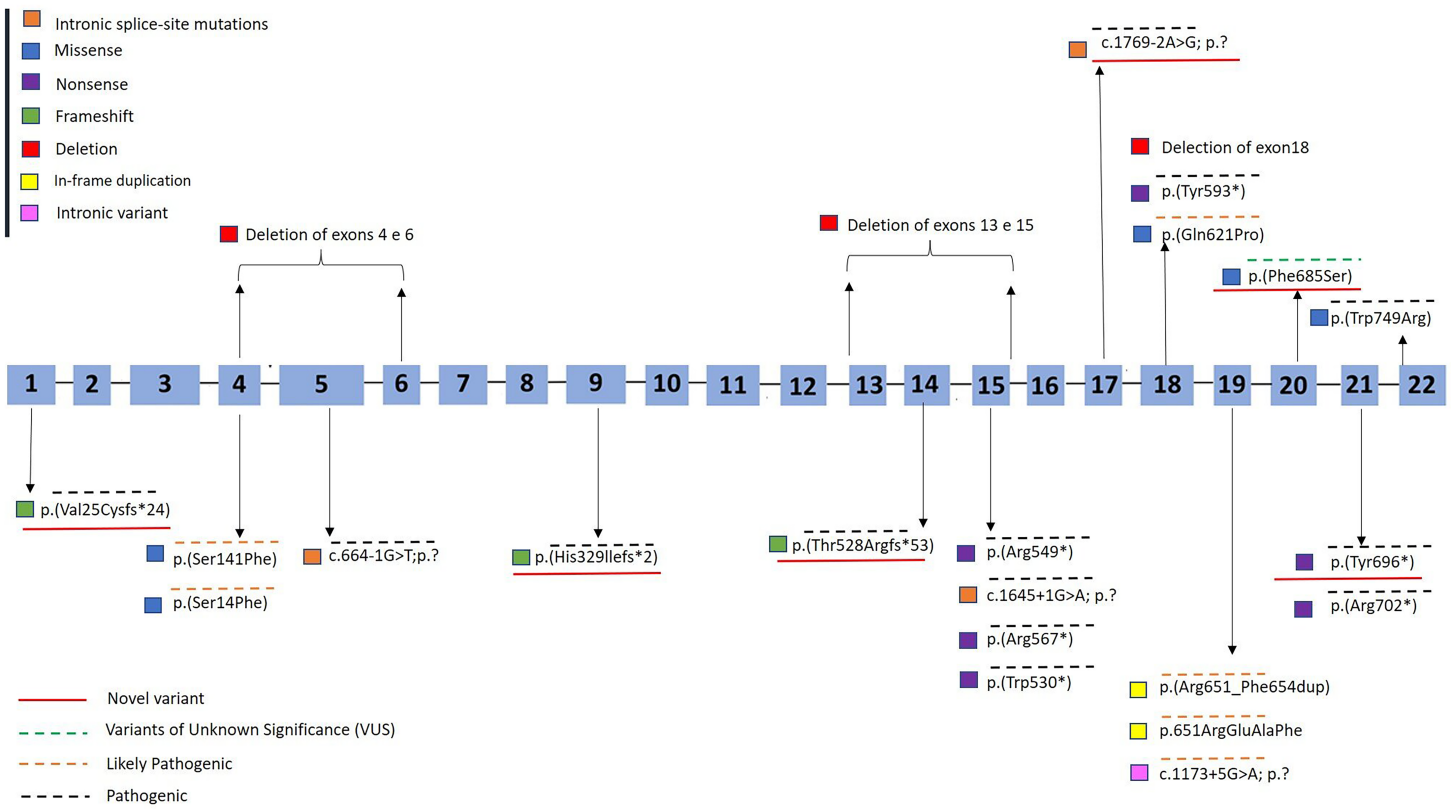
Diagram of the *PHEX* gene showing the variants detected in patients with X-linked hypophosphatemic rickets. The hatched blue bar represents the 22 exons of the gene. Novel variants were underlined in red. Variants found in this study are shown in yellow box. GenBank ID: NM_000444.6. Variants were classified according to recommendations from the consensus of the American College of Medical Genetics and Genomics and the Association for Molecular Pathology as pathogenic (overlined in black dashed line), likely pathogenic (overlined in orange dashed line), or variants of unknown significance (VUS) (13) (overlined in green dashed line).

**Figure 2:**
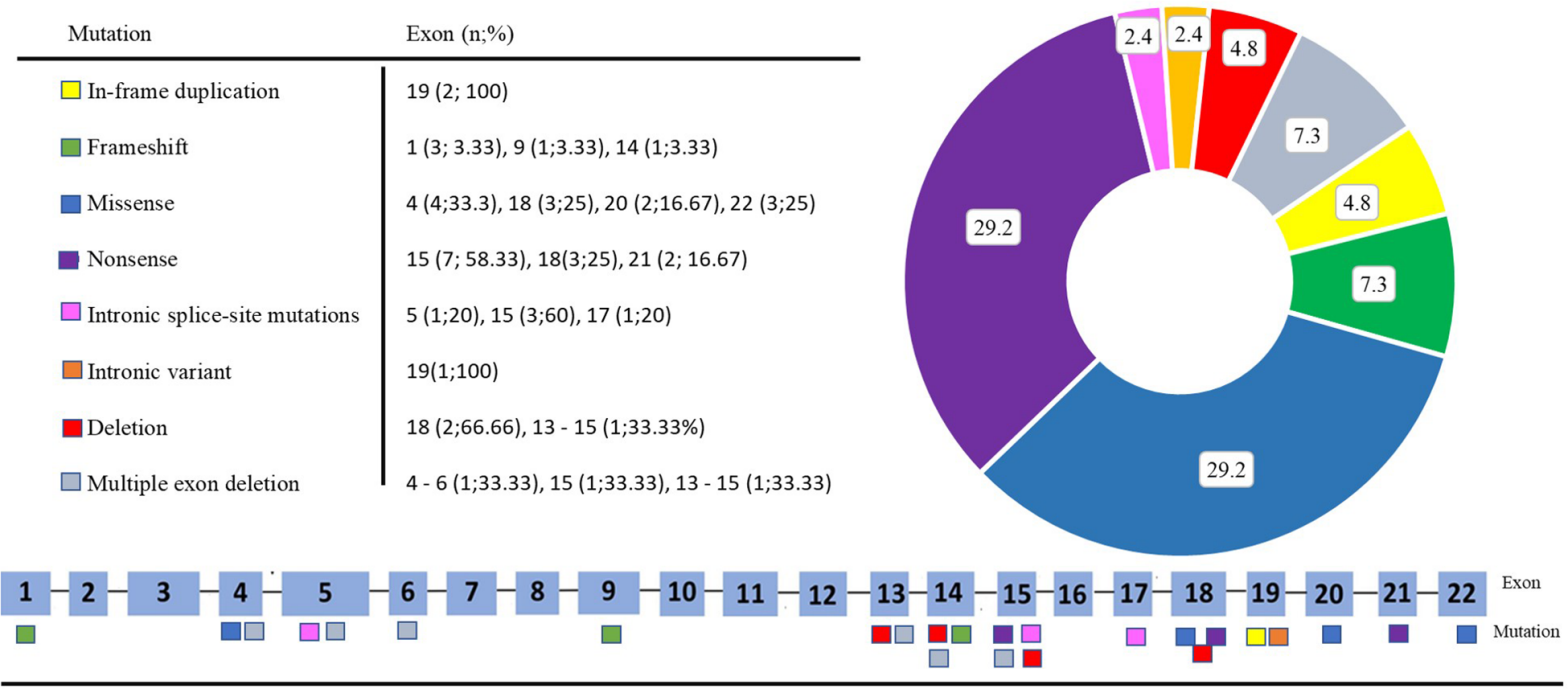
Distribution by type and exon of each variant.

**Figure 3:**
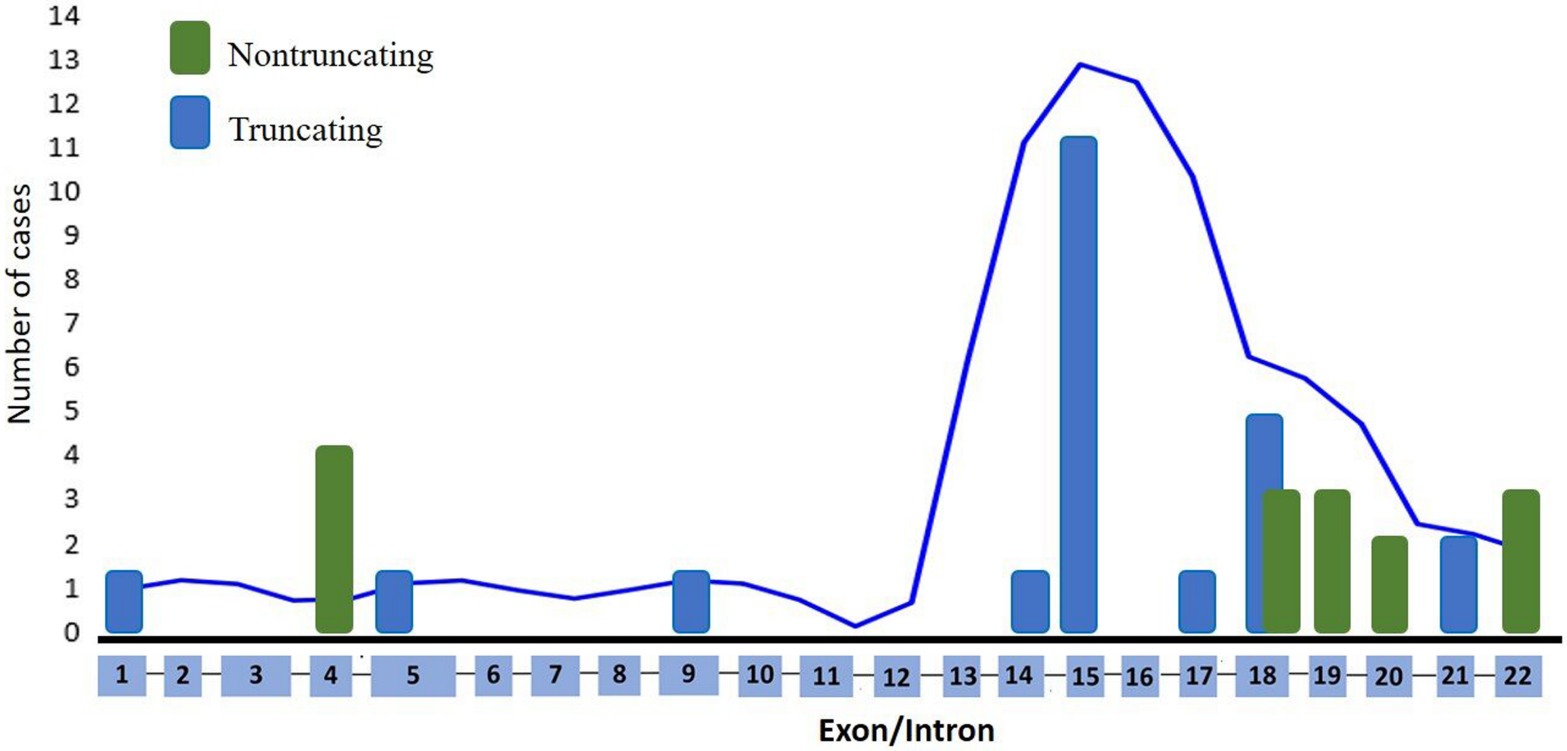
Distribution of *PHEX* variants found in this study.

Figure 1 presents the distribution of all identified variants. A total of 23 different *PHEX* variants were identified in 41 patients. Six novel variants were identified among seven patients, consisting of three frameshift variants, two missense variants, one canonical intronic splice-site variant, and one nonsense variant (Table 2). The detection along the *PHEX* gene showed that 15 variants are located from exon 15 to exon 22, whereas only seven variants are in other portions (Supplementary Figure S3). Among all the patients, truncating and non-truncating variants were found in 29 and 12 patients, respectively. Missense variants corresponded to 29.3% of the patients, and nonsense variants corresponded to 29.7% (Figure 2). In total, 63.4% of the variants were previously reported as deleterious (Figure 3). The most frequent mutation occurred in the region 208 (21.9%; 9/41 cases), with five cases in the position chrX:22, 208, and 619 (12.2%; 5/41 cases).

**Table 2:** Novel *PHEX* variants found in this study.

cDNA mutation^a^	Novel variants	Classification	Variant site	*In silico* prediction^b^
c.1582_1583delAC	p.(Thr528Argfs*53)	Frameshift	Exon 14	Deleterious
c.2088C >A	p.(Tyr696*)	Nonsense	Exon 21	Deleterious
c.2054T >C	p.(Phe685Ser)	Missense^c^	Exon 20	Deleterious
c.985delC	p.(His329llefs*2)	Frameshift	Exon 9	Deleterious
c.1769–2ª >G	p.?	Canonical intronic splice-site variants	Exon 17	Deleterious
c.70_74delGTCGT	p.(Val25Cysfs*24)	Frameshift	Exon 1	Deleterious

^a^ cDNA, complementary DNA (Ref Seq: NM_000444.6). Six novel variants in the PHEX gene were detected in seven patients.

^b^ *In silico* predictors used: missenses: SIFT, MutPred, MutationTaster, CAD-score variants; splice-site: MutationTaster, splice AI, and Human Splicing Finder; frameshift and nonsense: MutationTaster and CAD-score variants.

^c^ Novel variant [p.(Phe685Ser)] was identified in two patients.

Variants of unknown significance occurred in two cases in this cohort, both of which were novel missense variants [p.(Phe685Ser)]. Likely pathogenic variants were observed in 24.3% of the cases (10/41) (Figure 1).

### 3.3. Genotype–phenotype correlation findings in XLH patients

Initial stature, BMI, and related *Z*-scores did not show any significant difference according to protein domain regions (N-terminal half or in the C-terminal half of the *PHEX* protein) (*P *> 0.05) (Supplementary Table S1). The median initial serum concentrations of calcium, phosphorus, PTH, and alkaline phosphatase did not show significant differences among patients with protein domains located in the N-terminal or C-terminal regions, as well as with different pathogenicity and chromosome positions (*P *> 0.05) (Supplementary Table S2). XLH patients with a family history presented higher baseline concentrations of alkaline phosphatase (*P *= 0.03) (Supplementary Table S3). To evaluate the genotype–phenotype correlation, the patients were grouped into non-truncating (*n* = 12) and truncating (*n* = 29) variant groups according to their genotypes. No differences were found comparing gender and variant type (non-truncating vs. truncating) (Supplementary Table S4).

We observed a significant and positive correlation between the interpatellar distance and the anterior and posterior intercondylar distances (Supplementary Figure S4). However, no significant correlation was found between interpatellar or intercondylar distances and protein domain regions (*P *> 0.05) (Supplementary Table S5). Similarly, no correlation was found when comparing the initial serum concentrations of calcium, phosphorus, PTH, and alkaline phosphatase with the interpatellar distance or with the anterior or posterior intercondylar distance (Supplementary Figure S5).

Among XLH patients with bone deformity, 57.7% of the patients were referred to orthopedic surgery. However, the referral for orthopedic surgery was not significantly influenced by protein domain regions (*P *= 0.15) (Supplementary Table S6). There was also no significant correlation found between gender and the need for orthopedic surgery (*P *= 0.09). Notably, patients who underwent orthopedic surgery due to bone deformity exhibited significantly higher concentrations of PTH [72.05 pg/mL (52.7–79.57)] compared with those who did not require surgery [40.65 pg/mL (28.9–63.2), *P* = 0.05] (Supplementary Table S7). Furthermore, patients with variants leading to a putative truncation of the *PHEX* protein did not display a more severe phenotype when compared with patients with non-truncating variants. In addition, the outcome of orthopedic surgery was similar between patients with truncating and non-truncating variants (*P *= 0.23) (Supplementary Table S7). All reported variants are presented in Supplementary Table S8.

## 4. Discussion

This study aims to identify a correlation between phenotype and genotype by providing a current description of the phenotypic characteristics of the largest cohort of Brazilian XLH patients. However, no relevant correlation between initial clinical characteristic and type of variant were found in this cohort. Similar findings were previously reported, suggesting the absence of genotype–phenotype relationship in XLH caused by variants in the *PHEX* gene (8, 23, 24). Our survey comprised 41 children diagnosed with XLH in a center with experience in treating XLH over a period of 48 years. Based on the São Paulo state population, this number is lower than expected, indicating that the disease is either underdiagnosed or often treated in less specialized centers (25).

All types of variants were found in this cohort, and six of them were novel. The nonsense variant represented 26% of the causative variants found in this study. One nonsense variant was novel, and five variants had been previously described. A total of 23 different variants were distributed across the gene, and these variants have affected most exons or their adjacent introns (18). A major mutation density was found to be present from exon 15 to exon 22, corroborating with the findings of other studies (8, 26–28). Thus, the distribution in our cohort presents a lower mutation density in the first part of the gene according to *PHEX* mutation database (28). The clinical manifestations of XLH exhibited comparable characteristics irrespective of protein domain regions. An explanation for this could be that the genotype does not predict the phenotype (29). In addition, the laboratory biomarkers were not strictly dependent on the type or site location of the variant. Popowska et al. (27) found no relation between the severity of clinical symptoms and variant location. In concordance to that, no significant correlations were observed between the novel variant type and the serum concentrations of phosphate, PTH, or alkaline phosphatase at diagnosis, although one study reported that patients with truncating variants had lower serum phosphate concentration compared with patients without truncating variants (8). In addition, a cohort of 21 patients from the Norwegian pediatric population showed no significant differences in growth, dental involvement, persistent bowing, or development of nephrocalcinosis between the variant status groups (30). A multicenter study conducted in Australia and in the Unites States found no correlation between the severity of skeletal manifestations and the type or location of *PHEX* variants (7). However, the association between more severe skeletal disease and truncating variants reached no statistical difference in this relatively small sample size (7). XLH is a very heterogeneous disease in terms of clinical manifestation, along with the phenotypical variability of the disease that is dependent on the characteristics of the studied population even among patients with truncating variant.

Intercondylar and interpatellar distances were previously described (29). An intercondylar distance has been reported to be a predictor of adequacy of the treatment response (29), and the results of our study showed that the intercondylar distance after treatment has significantly reduced compared with the observed distance prior to treatment, which was 4.5 cm (3.0–6.0) vs. 1.5 cm (0–3.3); *P* < 0.05) (29). Our study is the first to evaluate the correlation between interpatellar or intercondylar distance and genotype; however, no significant correlation was identified. In fact, a huge phenotype variation between individuals with the same genotype was described, particularly regarding the skeletal phenotype (7, 31).

Osteotomy was required in 57% of our patients at some point of life, highlighting the negative impact of the disease on the mobility and quality of life of those affected. Our results are similar to the findings of other studies, reporting that 24%–65% of XLH patients will require surgical intervention for lower limb deformity (7, 23, 32). According to the data from an Italian center where 175 patients were followed up from 1998 to 2017, XLH was diagnosed before the age of 1 year in 11% of the cases, and it was diagnosed between 1 and 5 years in 50% of the cases (25). Clinically apparent bone deformities were present in 95% of the patients (25). In our study, diagnosis was delayed in most of the patients with 72% of the cases diagnosed in the first 5 years of life. According to Morey et al. (8), significantly higher pretreatment concentrations of PTH were observed in patients requiring orthopedic surgery due to bone deformity. Hyperparathyroidism is a complicating factor in XLH adult patients and represents a negative prognostic index, which indicates a higher prevalence of lower limb deformities (33).

This study had a number of limitations commonly encountered in retrospective studies, which is being highly dependent on observations being recorded by the treating clinicians and on some records without complete information, implying that we could not ensure uniform collection of information from the clinical, biochemical, and radiological examinations. In addition, some patients were diagnosed at later ages, which could have worsened their bone phenotype and may have affected the measurement of interpatellar and intercondylar distances. Finally, the study is limited by the size of a single-center cohort.

In conclusion, identifying of variants in the *PHEX* gene seems to have a limited prognostic value, since no significant correlation was observed between genotype and phenotype in our study, as well as in other studies. On the other hand, genetic testing is useful to allow identifying affected individuals early and providing adequate treatment. This study identified six new variants in the *PHEX* gene, and it contributes to increasing our knowledge with regard to the types of variants related to XLH. Future considerations regarding the genotype and the precise treatment based on the pathophysiology of this condition will have the potential to improve the quality of life, social inclusion, genetic counseling, and the reduction of bone deformities and surgery requirement of the patients.

## Data Availability

The original contributions presented in the study are included in the article/**Supplementary Material**, further inquiries can be directed to the corresponding author.

## References

[1] Haffner D Emma F Eastwood DM Duplan MB Bacchetta J Schnabel D Clinical practice recommendations for the diagnosis and management of X-linked hypophosphataemia. Nat Rev Nephrol. (2019) 15(7):435–55. 10.1038/s41581-019-0152-5 31068690 PMC7136170

[2] Chandran M Chng CL Zhao Y Bee YM Phua LY Clarke BL. Novel PHEX gene mutation associated with X linked hypophosphatemic rickets. Nephron Physiol. (2010) 116(3):17–21. 10.1159/000319318 20664300

[3] Tenenhouse HS. X-linked hypophosphataemia: a homologous disorder in humans and mice. Nephrol Dial Transplant. (1999) 14(2):333–41. 10.1093/ndt/14.2.333 10069185

[4] Beck-Nielsen SS Brock-Jacobsen B Gram J Brixen K Jensen TK. Incidence and prevalence of nutritional and hereditary rickets in Southern Denmark. Eur J Endocrinol. (2009) 160(3):491–7. 10.1530/EJE-08-0818 19095780

[5] Rowe PS Goulding JN Francis F Oudet C Econs MJ Hanauer A The gene for X-linked hypophosphatemic rickets maps to 200–300 kb region in Xp22.1, and is located on a single YAC containing a putative vitamin D response element (VDRE). Hum Genet. (1996) 97:345–52. 10.1007/BF02185769 8786079

[6] Francis F Strom TM Hennig S Böddrich A Lorenz B Brandau O Genomic organization of the human PEX gene mutated in X-linked dominant hypophosphatemic rickets. Genome Res. (1997) 7(6):573–85. 10.1101/gr.7.6.573 9199930

[7] Holm IA Nelson AE Robinson BG Mason RS Marsh DJ Cowell CT Mutational analysis and genotype-phenotype correlation of the PHEX gene in X-linked hypophosphatemic rickets. J Clin Endocrinol Metab. (2001) 86:3889–99. 10.1210/jcem.86.8.7761 11502829

[8] Morey M Castro-Feijoo L Barreiro J Cabanas P Pombo M Gil M Genetic diagnosis of X-linked dominant hypophosphatemic rickets in a cohort study: tubular reabsorption of phosphate and 1,25(OH)2D serum levels are associated with PHEX mutation type. BMC Med Genet. (2011) 12:116. 10.1186/1471-2350-12-116 21902834 PMC3189111

[9] Park PG Lim SH Lee H Ahn YH Cheong HI Kang HG. Genotype and phenotype analysis in X-linked hypophosphatemia. Front Pediatr. (2021) 9:699767. 10.3389/fped.2021.699767 34434907 PMC8382157

[10] Song HR Park JW Cho DY Yang JH Yoon HR Jung SC. PHEX Gene mutations and genotype-phenotype analysis of Korean patients with hypophosphatemic rickets. J Korean Med Sci. (2007) 22:981–6. 10.3346/jkms.2007.22.6.981 18162710 PMC2694264

[11] Ogden CL Kuczmarski RJ Flegal KM Mei Z Guo S Wei R Centers for Disease Control and Prevention 2000 growth charts for the United States: improvements to the 1977 National Center for Health Statistics version. Pediatrics. (2002) 109(1):45–60. 10.1542/peds.109.1.45 11773541

[12] Aziz N Zhao Q Bry L Driscoll DK Funke B Gibson JS College of American Pathologists’ laboratory standards for next-generation sequencing clinical tests. Arch Pathol Labo Med. (2015) 139(4):481–93. 10.5858/arpa.2014-0250-CP 25152313

[13] Richards S Aziz N Bale S Bick D Das S Gastier-Foster J ACMG Laboratory quality assurance committee. Standards and guidelines for the interpretation of sequence variants: a joint consensus recommendation of the American College of Medical Genetics and Genomics and the Association for Molecular Pathology. Genet Med. (2015) 17(5):405–24. 10.1038/gim.2015.30 25741868 PMC4544753

[14] Gudmundsson S Singer-Berk M Watts NA Phu W Goodrich JK Solomonson M Genome Aggregation Database Consortium, Variant interpretation using population databases: lessons from gnomAD. Hum Mutat. (2022) 43(8):1012–30. 10.1002/humu.24309 34859531 PMC9160216

[15] Desmet FO Hamroun D Lalande M Collod-Béroud G Claustres M Béroud C. Human splicing finder: an online bioinformatics tool to predict splicing signals. Nucleic Acids Res. (2009) 37(9):e67. 10.1093/nar/gkp215 19339519 PMC2685110

[16] Tian Y Pesaran T Chamberlin A Fenwick RB Li S Gau CL REVEL and BayesDel outperform other in silico meta-predictors for clinical variant classification. Sci Rep. (2019) 9(1):12752. 10.1038/s41598-019-49224-8 31484976 PMC6726608

[17] de Sainte Agathe JM Filser M Isidor B Besnard T Gueguen P Perrin A SpliceAI-visual: a free online tool to improve SpliceAI splicing variant interpretation. Hum Genomics. (2023) 17(1):7. 10.1186/s40246-023-00451-1 36765386 PMC9912651

[18] Landrum MJ Lee JM Benson M Brown GR Chao C Chitipiralla S ClinVar: improving access to variant interpretations and supporting evidence. Nucleic Acids Res. (2018) 46(D1):D1062–7. 10.1093/nar/gkx1153 29165669 PMC5753237

[19] Stenson PD Mort M Ball EV Chapman M Evans K Azevedo L The human gene mutation database (HGMD®): optimizing its use in a clinical diagnostic or research setting. Hum Genet. (2020) 139(10):1197–207. 10.1007/s00439-020-02199-3 32596782 PMC7497289

[20] Sarafrazi S Daugherty SC Miller N Boada P Carpenter TO Chunn L Novel PHEX gene locus-specific database: comprehensive characterization of vast number of variants associated with X-linked hypophosphatemia (XLH). Hum Mutat. (2022) 43(2):143–57. 10.1002/humu.24296 34806794 PMC9299612

[21] Chunn LM Nefcy DC Scouten RW Tarpey RP Chauhan G Lim MS Mastermind: a comprehensive genomic association search engine for empirical evidence curation and genetic variant interpretation. Front Genet. (2020) 11:577152. 10.3389/fgene.2020.577152 33281875 PMC7691534

[22] Cho HY Lee BH Kang JH Ha IS Cheong HI Choi Y. A clinical and molecular genetic study of hypophosphatemic rickets in children. Pediatr Res. (2005) 58(2):329–33. 10.1203/01.PDR.0000169983.40758.7B 16055933

[23] Chesher D Oddy M Darbar U Sayal P Casey A Ryan A Outcome of adult patients with X-linked hypophosphatemia caused by PHEX gene mutations. J Inherit Metab Dis. (2018) 41(5):865–76. 10.1007/s10545-018-0147-6 29460029 PMC6133187

[24] Gaucher C Walrant-Debray O Nguyen TM Esterle L Garabedian M Jehan F. PHEX Analysis in 118 pedigrees reveals new genetic clues in hypophosphatemic rickets. Hum Genet. (2009) 125:401–11. 10.1007/s00439-009-0631-z 19219621

[25] Emma F Cappa M Antoniazzi F Bianchi M Chiodini I Vainicher CE X-linked hypophosphatemic rickets: an Italian experts’ opinion survey. Ital J Pediatr. (2019) 45(1):67. 10.1186/s13052-019-0654-6 31151476 PMC6545008

[26] Sabbagh Y Jones AO. Tenenhouse HS: PHEXdb, a locus-specific database for mutations causing X-linked hypophosphatemia. Hum Mutat. (2000) 16(1):1–6. 10.1002/1098-1004(200007)16:1<1::AID-HUMU1>3.0.CO;2-J 10874297

[27] Sant’ Ana I Torrini R Alves Coelho MC Cantoni J Madeira M Ribeiro M. X-linked hypophosphatemic rickets: description of seven new variants in patients followed up in reference hospitals in Rio de Janeiro. Mol Genet Genomic Med. (2022) 10(6):e1941. 10.1002/mgg3.1941 35384411 PMC9184672

[28] Popowska E Pronicka E Sułek A Jurkiewicz D Rowińska E Sykut-Cegielska J X-linked hypophosphatemia in Polish patients. 2. Analysis of clinical features and genotype-phenotype correlation. J Appl Genet. (2001) 42(1):73–88. 14564066

[29] Wei LY Gong CX Cao BY Li XQ Liang XJ Li WJ [Genetic and clinical analysis of X-linked hypophosphatemic rickets]. Zhonghua Er Ke Za Zhi. (2021) 59(8):678–83. 34333921 10.3760/cma.j.cn112140-20210311-00201

[30] Rafaelsen S Johansson S Ræder H Bjerknes R. Hereditary hypophosphatemia in Norway: a retrospective population-based study of genotypes, phenotypes, and treatment complications. Eur J Endocrinol. (2016) 174(2):125–36. 10.1530/EJE-15-0515 26543054 PMC4674593

[31] Econs MJ Friedman NE Rowe PS Speer MC Francis F Strom TM A PHEX gene mutation is responsible for adult-onset vitamin D-resistant hypophosphatemic osteomalacia: evidence that the disorder is not a distinct entity from X-linked hypophosphatemic rickets. J Clin Endocrinol Metab. (1998) 83(10):3459–62. 9768646 10.1210/jcem.83.10.5167

[32] Evans GA Arulanantham K Gage JR. Primary hypophosphatemic rickets. Effect of oral phosphate and vitamin D on growth and surgical treatment. J Bone Joint Surg Am. (1980) 62:1130–8. 10.2106/00004623-198062070-00010 6253500

[33] Lecoq AL Chaumet-Riffaud P Blanchard A Dupeux M Rothenbuhler A Lambert B Hyperparathyroidism in patients with X-linked hypophosphatemia. J Bone Miner Res. (2020) 35(7):1263–73. 10.1002/jbmr.3992 32101626

