# Supplementary material for: A genetic study of a Brazilian cohort of patients with X-linked hypophosphatemia reveals no correlation between genotype and phenotype
Source: Front Pediatr. 2023 Sep 19;11:1215952. doi: 10.3389/fped.2023.1215952 (PMC10546205; doi:10.3389/fped.2023.1215952)
Supplement: Supplementary file 1 [file Datasheet1.pdf]

# Supplementary Material

Borghi M et al.

**Table of contents**


| Supplementary Files | Tables and Figure Captions | Page |
|---|---|---|
| **Supplementary Figure 1** | Measurements of interpatellar distance (A), anterior (B), and posterior (C) intercondylar distance. | **2** |
| **Supplementary Figure 2** | Families' pedigrees. The black symbols signify individuals affected by the condition, while the open symbols denote those unaffected. The circles and squares indicate female and male, respectively. The arrows serve to designate the proband in the families. | **2** |
| **Supplementary Table 1** | Relationship between protein domain and initial height, initial Z-height, initial BMI ($kg/m^2$), initial Z-BMI. | **3** |
| **Supplementary Figure 3** | Distribution of mutations by exon. On the left, there is a dashed curve representing the density estimate by Kernel. On the right, the mutation frequencies in each exon are shown. | **3** |
| **Supplementary Table 2** | Genotype-phenotype correlations in patients with a *PHEX* gene mutation: Protein domain vs. initial values of calcium (mg/dL), phosphorus (mg/dL), alkaline phosphatase (UI/L) and parathyroid hormone - PTH (pg/mL). | **4** |
| **Supplementary Table 3** | Genotype-phenotype correlations in patients with a *PHEX* gene mutation in terms of initial values of calcium (mg/dL), phosphorus (mg/dL), alkaline phosphatase (UI/L) and parathyroid hormone - PTH (pg/mL). | **4** |
| **Supplementary Table 4** | Genotype-phenotype correlations in patients with a *PHEX g*ene mutation in terms of family history. | **5** |
| **Supplementary Table 5** | Genotype-phenotype correlations in patients with a *PHEX* gene mutation in terms of gender and mutation type. | **5** |
| **Supplementary Figure 4** | Correlation between the interpatellar distance(a) and the anterior(b) and posterior intercondylar (c) distance (cm). Spearman correlation. | **5** |
| **Supplementary Table 6** | Relationship between location of the *PHEX* gene mutation and the interpatellar distance, the anterior, and posterior intercondylar distance. | **6** |
| **Supplementary Figure 5** | Correction matrix between interpatellar, anterior intercondylar and posterior intercondylar distances and initial values of calcium (mg/dL), phosphorus (mg/dL), alkaline phosphatase (UI/L) and parathyroid hormone - PTH (pg/mL). Each table cell shows the correlation between the two factors. Spearman correlation ($P$>0.05). | **6** |
| **Supplementary Table 7** | Analysis of genotypic predictors in relation to performing an orthopedic surgical procedure. | **7** |
| **Supplementary Table 8** | Reported variants (Detailed genotypic information of patients with *PHEX* variants). | **7** |

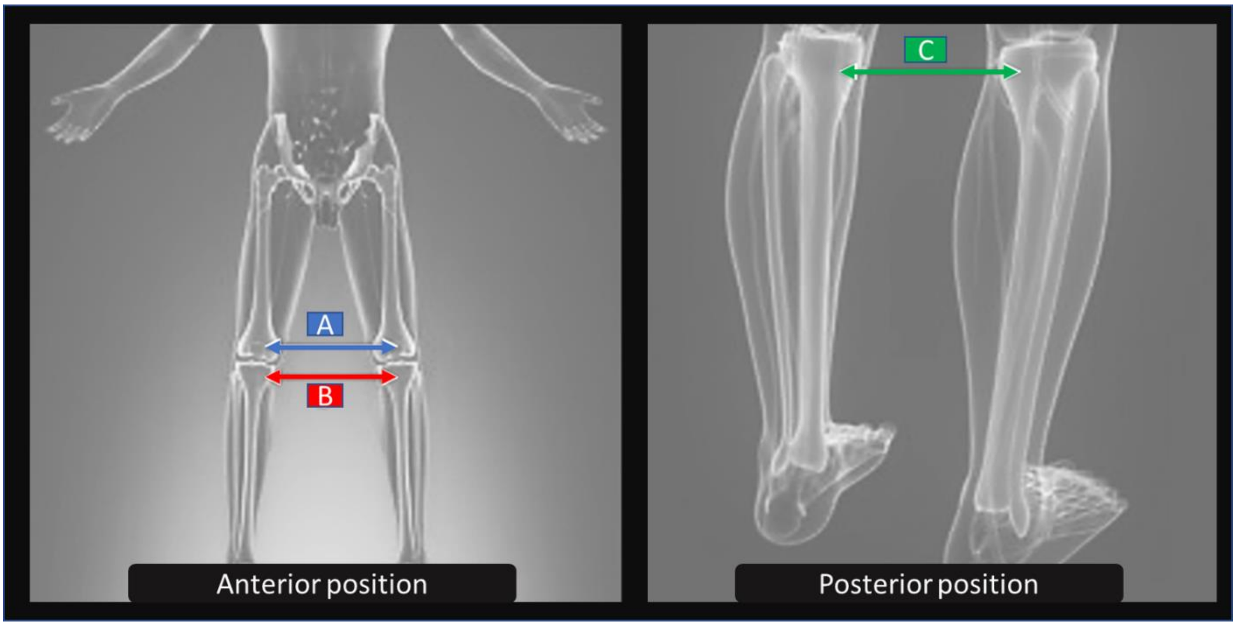


**Supplementary Figure 1.** Measurements of interpatellar distance (A), anterior (B), and posterior (C) intercondylar distance.

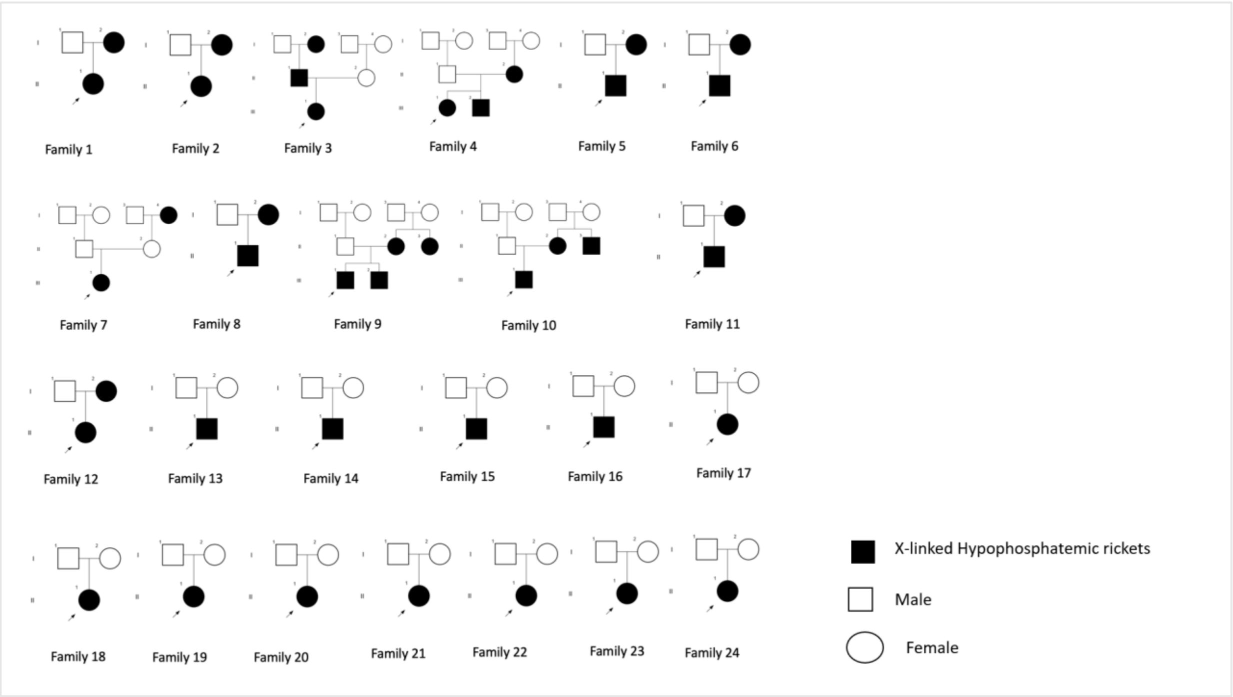


**Supplementary Figure 2.** Families' pedigrees. The black symbols signify individuals affected by the condition, while the open symbols denote those unaffected. The circles and squares indicate female and male, respectively. The arrows serve to designate the proband in the families.

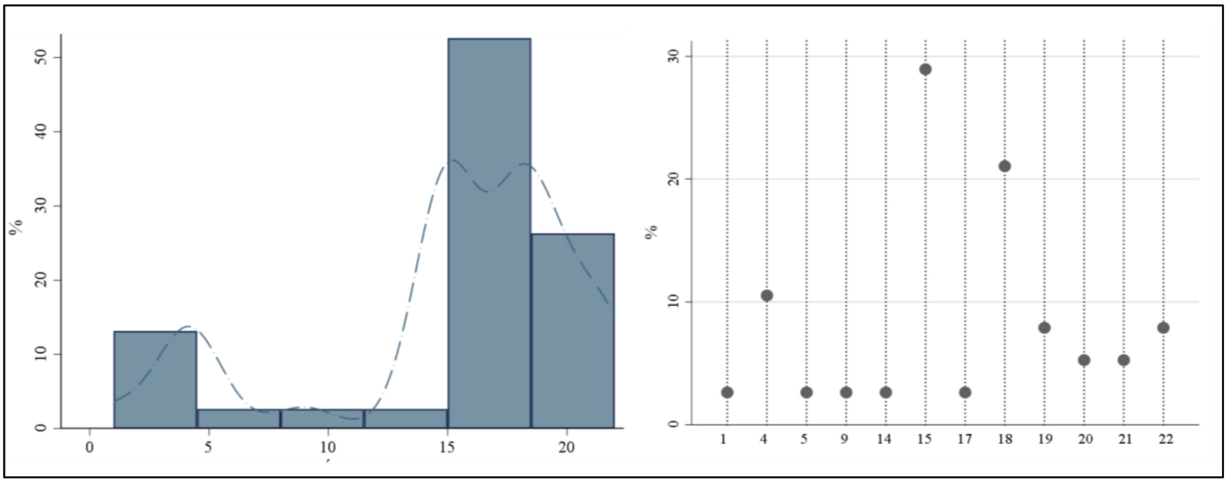


**Supplementary Figure 3.** Distribution of variants by exon. On the left, there is a dashed curve representing kernel density estimation. On the right, the mutation frequencies in each exon are shown.

**Supplementary Table 1.** Relationship between protein domain and initial height, initial Z-height, initial BMI (kg/m$^2$), initial Z-BMI.

| | **Protein Domain** | | | P-value* |
|---|---|---|---|---|
| | C-terminal half | N-terminal half | Other** | |
| Initial height (cm) | 87.5(81-99.3) | 105(79.3-119.1) | 80.7 | 0.36 |
| Initial Z-height (cm) | -2.06(-2.55- -1.38) | -1.71(-3.83- -0.48) | -3.4 | 0.43 |
| Initial BMI (kg/m$^2$) | 17.42(16.2-18.8) | 18.1(17-21.06) | 18.43 | 0.65 |
| Initial Z-BMI (kg/m$^2$) | 1.01(0.36-1.38) | 1.39(0.19-1.79) | 1.55 | 0.52 |

Values expressed as Median (25-75% percentile). *Kruskal Wallis analysis of variance.** Deletions and variants located in the last codon of the gene.

**Supplementary Table 2.** Genotype-phenotype correlations in patients with a *PHEX* gene mutation: Protein domain vs. initial values of calcium (mg/dL), phosphorus (mg/dL), alkaline phosphatase (UI/L) and parathyroid hormone - PTH (pg/mL).

| | **Protein Domain** | | | P-value* |
|---|---|---|---|---|
| | C-terminal half | N-terminal half | Other** | |
| Calcium (mg/dL) | 9.7(9.2-9.9) | 9.8(9.55-10.5) | 8.3 | 0.15 |
| Phosphorus (mg/dL) | 2.65(2.4-2.82) | 2.8(1.85-3.35) | 2.1 | 0.37 |
| Alkaline phosphatase (UI/L) | 1102(485.5-1436) | 423(204.5-1251.5) | 332 | 0.11 |
| PTH (pg/mL) | 47.9(30.52-67.8) | 83(76-90) | 76.1 | 0.06 |

Values expressed as median (percentile 25-75%). *Analyses of variance were performed with the Kruskal-Wallis test. ** Deletions and variants located in the last codon of the gene.

**Supplementary Table 3.** Genotype-phenotype correlations in patients with a *PHEX* gene mutation in terms of family history.

| | **Family history** | | P-value* |
|---|---|---|---|
| | No | Yes | |
| Initial height (cm) | 92.05(84.3-104.15) | 93(77.45-108) | 0.73 |
| Initial Z-height (cm) | -1.78(-2.45- -0.44) | -2.38(-3.33 - -1.86) | 0.18 |
| Initial BMI (kg/m$^2$) | 17.98(16.50-19.12) | 18.2(16.36-18.8) | 0.96 |
| Initial Z-BMI (kg/m$^2$) | 1.4(0.51-1.94) | 1.35(0.42-1.38) | 0.21 |
| Calcium (mg/dL) | 9.6(9.3-10.3) | 9.8(9.37-9.9) | 0.81 |
| Phosphorus (mg/dL) | 2.4(2.32-2.8) | 2.65(2.1-2.9) | 0.79 |
| Alkaline phosphatase (UI/L) | 445.5(350-1041.7) | 1174(647.7-1609-25) | 0.03 |
| PTH (pg/mL) | 38.55(28.9-68.2) | 68.1(58.8-83) | 0.19 |

Values expressed as median (percentile 25-75%). Mann-Whitney test.

**Supplementary Table 4.** Genotype-phenotype correlations in patients with a *PHEX* gene mutation in terms of gender and mutation type.

| | **Types % (n)** | | P-value* |
|---|---|---|---|
| **Gender** | Truncating | Nontruncating | 0.25 |
| Female | 20 (68.97) | 6 (50) | |
| Male | 9 (31.03) | 6 (50) | |

Values expressed in % (n). *Chi-square test.

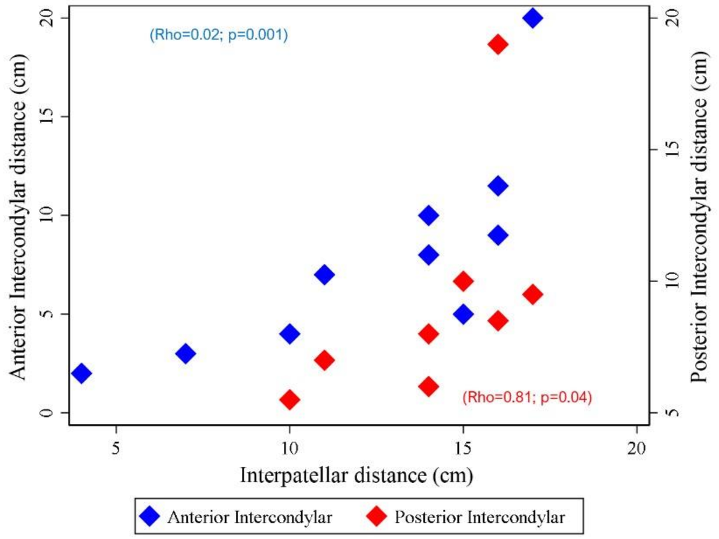


**Supplementary Figure 4.** Correlation between the interpatellar distance(a) and the anterior(b) and posterior intercondylar (c) distance (cm). Spearman correlation.

**Supplementary Table 5.** Relationship between protein domain and the interpatellar distance, the anterior, and posterior intercondylar distance.

| | **Protein domain** | | | P-value* |
|---|---|---|---|---|
| | C-terminal half | N-terminal half | Other* | |
| Interpatellar (cm) | 14(7-16) | NA | 14 | NA |
| Anterior intercondylar (cm) | 5(3-11.5) | 9.5 | 10 | 0.65 |
| Posterior intercondylar (cm) | 9.5(5.75-14.5) | NA | 8 | NA |

Values expressed as median (percentile 25-75%). *Analyses of variance were performed with the Kruskal-Wallis test. NA – Not applicable. ** Deletions and variants located in the last codon of the gene.

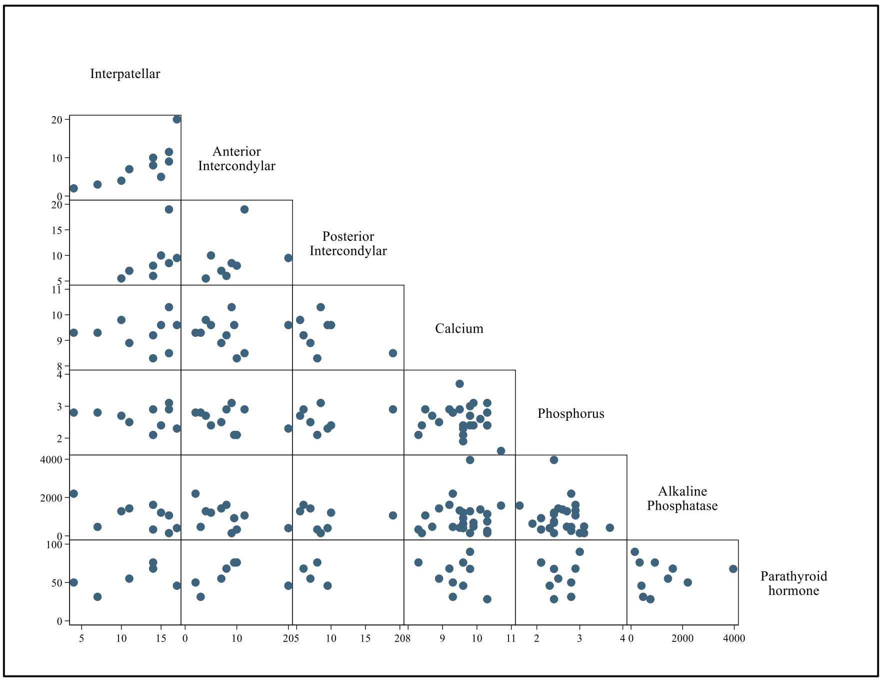


**Supplementary Figure 5.** Correction matrix between interpatellar, anterior intercondylar and posterior intercondylar distances and initial values of Calcium (mg/dL), Phosphorus (mg/dL), Alkaline phosphatase (UI/L) and Parathyroid hormone - PTH (pg/mL). Each table cell shows the correlation between the two factors. Spearman correlation for each correlation (P>0.05).

**Supplementary Table 6.** Analysis of genotypic predictors in relation to performing an orthopedic surgical procedure.

| Genotypic predictor | **Orthopedic surgical procedure** | | P-value* |
|---|---|---|---|
| | No % (n) | Yes % (n) | |
| **Ptotein domain** | | | 0.15 |
| C-Terminal | 90(9) | 53.85(7) | |
| N-Terminal | 10(1) | 30.77(4) | |
| Others | 0(0.00) | 15.38(2) | |
| **Type** | | | 0.23 |
| Truncating | 81.82 (9) | 60 (9) | |
| Nontruncating | 18.18 (2) | 40 (6) | |

Values expressed in % (n). *Chi-square test.

**Supplementary Table 7.** Analysis of Phenotype predictors in relation to performing an orthopedic surgical procedure.

| Phenotype predictors | **Orthopedic surgical procedure** | | P-value |
|---|---|---|---|
| | No % (n) | Yes % (n) | |
| **Gender** | | | 0.09 |
| Female | 72.73(8) | 40(6) | |
| Male | 27.27(3) | 60(9) | |
| **Family history** | | | 0.79 |
| No | 4 (36.36) | 5(41.67) | |
| Yes | 7(63.64) | 7(58.33) | |
| **Initial Calcium#** | 9.6 | 9.6 | 0.70 |
| **Initial Phosphorus#** | 2.7 | 2.6 | 0.69 |
| **Initial Z-height (cm)** | -0.6 | -0.37 | 0.80 |
| **Initial Z-BMI (kg/m2)** | -0.42 | 0.9 | 0.06 |
| **Alkaline phosphatase (UI/L)#** | 750 | 475 | 0.23 |
| **PTH (pg/mL)#** | 40.6 | 72.0 | 0.05 |

* Values expressed in % (n). Chi-square test. **Values expressed in median. Mann-Whitney test. #Calcium (mg/dL), phosphorus (mg/dL), alkaline phosphatase (UI/L) and parathyroid hormone - PTH (pg/mL) initials.

**Supplementary Table 8.** Reported variants (Detailed genotypic information of patients with *PHEX* mutation).

| Case | Gender | *PHEX* (Position) | Variant | Predicted effect | Variant site | ACMG classification[1] |
|---|---|---|---|---|---|---|
| 1 | M | chrX:22.266.065 | c.2245T>C | p.(Trp749Arg) | Exon 22 | Pathogenic PS1+PM2+PP3+PP4+PP5 |
| 2 | M | chrX:22.208.619 | c.1645C>T | p.(Arg549*) | Exon 15 | Pathogenic PVS1+PS3+PS4+PM2+PP4+PP5 |
| 3 | M | chrX:22.080.328-22.108.729 | | Delection Exon 4 - 6 | CNV | |
| 4 | F | chrX:22.208.620 | c.1645+1G>A | p.Arg549Cysfs*20 | Intron 15 | Pathogenic PVS1+PS3+PM2+PP4+PP5 |
| 5 | M | chrX:22.245.712 | c.2054T>C | p.(Phe685Ser) | Exon 20 | VUS PM2+PP3+PP4+PP5 |
| 6 | M | chrX:22.196.488 | c.1582_1583delAC | p.(Thr528Argfs*53) | Exon 14 | Pathogenic PVS1+PM2+PP4 |
| 7 | F | chrX:22.263.467 | c.2088C>A | p.(Tyr696*)2 | Exon 21 | Pathogenic PVS1+PM2+PM6+PP4 |
| 8 | F | chrX:22.266.065 | c.2245T>C | p.(Trp749Arg) | Exon 22 | Pathogenic PS1+PM2+PP3+PP4+PP5 |
| 9 | F | chrX:22.239.642-22.240.038 | | Deletion Exon 18 | 18 | |
| 10 | F | chrX:22.094.578 | c.422C>T | p.(Ser141Phe)3 | Exon 4 | Likely Pathogenic PM2+PP3+PP4+PP5 |
| 11 | M | chrX:22.094.578 | c.422C>T | p.(Ser141Phe) | Exon 4 | Likely Pathogenic PM2+PP3+PP4+PP5 |
| 12 | F | chrX:22.245.712 | c.2054T>C | p.(Phe685Ser) | Exon 20 | VUS PM2+PP3+PP4++PP5 |
| 13 | F | chrX:22.208.620 | c.1645+1G>A | p.Arg549Cysfs*20 | Intron 15 | Pathogenic PVS1+PS3+PM2+PP4+PP5 |

| | | | | | | |
|---|---|---|---|---|---|---|
| 14 | M | chrX:22.239.642-22.240.038 | | Deletion Exon 18 | 18 | |
| 15 | F | chrX:22.208.620 | c.1645+1G>A | p.Arg549Cysfs*20 | Intron 15 | Pathogenic PVS1+PS3+PM2+PP4+PP5 |
| 16 | F | chrX:22.231.074 | c.1699C>T | p.(Arg567*)4 | Exon 15 | Pathogenic PVS1+PS4+PS3+PM2+PP4+PP5 |
| 17 | M | chrX:22.239.740 | c.1779T>A | p.(Tyr593*) | Exon 18 | Pathogenic PVS1+PM2+PM6+PP4+PP5 |
| 18 | F | chrX:22.208.619 | c.1645C>T | p.(Arg549*) | Exon 15 | Pathogenic PVS1+PS3+PS4+PM2+PP4+PP5 |
| 19 | F | chrX:22.094.578 | c.422C>T | p.(Ser141Phe) | Exon 4 | Likely Pathogenic PM2+PM5+PP3+PP4+PP5 |
| 20 | F | chrX:22.239.740 | c.1779T>A | p.(Tyr593*) | Exon 18 | Pathogenic PVS1+PM2+PM6+PP4+PP5 |
| 21 | F | chrX:22.117.170-22.117171 | c.985delC | p.(His329llefs*2) | Exon 9 | Pathogenic PVS1+PM2+ PP4 |
| 22 | M | chrX:22.263.483 | c.2104C>T | p.(Arg702*) | Exon 21 | Pathogenic PVS1+PS4+PM2+PM6+PP4+PP5 |
| 23 | F | chrX:22.231.074 | c.1699C>T | p.(Arg567*) | Exon 15 | Pathogenic PVS1+PS4+PS3+PM2+PP4+PP5 |
| 24 | F | chrX:22.244.610 | c.1951_1962dupCGGGAAGCTTTT | p.(Arg651_Phe654dup) | Exon 19 | Likely Pathogenic |
| 25 | F | chrX:22.208.619 | c.1645C>T | p.(Arg549*) | Exon 15 | Pathogenic PVS1+PS3+PS4+PM2+PP4+PP5 |
| 26 | M | chrX:22.244.610 | c.1951_1962dupCGGGAAGCTTTT | p.651ArgGluAlaPhe | Exon 19 | Likely Pathogenic PM2+PM4+PM6+PP4 |
| 27 | F | chrX:22.208.564 | c.1590G>A | p.(Trp530*) | Exon 15 | Pathogenic PVS1+PM2+PP4 |
| 28 | F | chrX:22.186.349-22.208.736 | | Deletion Exon 13 - 15 | | |
| 29 | F | chrX:22.208.619 | c.1645C>T | p.(Arg549*) | Exon 15 | Pathogenic PVS1+PS3+PS4+PM2+PP4+PP5 |
| 30 | M | chrX:22.094.578 | c.422C>T | p.(Ser141Phe) | Exon 4 | Likely Pathogenic PM2+PM5+PP3+PP4+PP5 |
| 31 | M | chrX:22.239.823 | c.1862A>C | p.(Gln621Pro) | Exon 18 | Likely Pathogenic |
| 32 | F | chrX:22.186.349-22.208.736 | | Deletion Exon 13 - 15 | | |
| 33 | F | chrX:22.208.619 | c.1645C>T | p.(Arg549*) | Exon 15 | Pathogenic PVS1+PS3+PS4+PM2+PP4+PP5 |
| 34 | M | chrX:22.266.065 | c.2245T>C | p.(Trp749Arg) | Exon 22 | Pathogenic PS1+PM2+PP3+PP4+PP5 |
| 35 | M | chrX:22.239.740 | c.1779T>A | p.(Tyr593*) | Exon 18 | Pathogenic PVS1+PM2+PM6+PP4+PP5 |
| 36 | F | chrX:22.239.823 | c.1862A>C | p.(Gln621Pro) | Exon 18 | Likely Pathogenic PM2+PS4+PP3+PP4 |
| 37 | F | chrX:22.239.823 | c.1862A>C | p.(Gln621Pro) | Exon 18 | Likely Pathogenic PM2+PS4+PP3+PP4 |
| 38 | M | chrX:22.129.683 | c.1173+5G>A | p.?7 | Intron 19 | Likely Pathogenic PM2+PM6+PP3+PP4+PP5 |
| 39 | F | chrX:22.239.728 | c.1769-2A>G | p.? | Intron 17 | Pathogenic PVS1+PM2+PP5 |
| 40 | F | chrX:22.108.546 | c.664-1G>T | p.? | Intron 5 | Pathogenic PVS1+PM2+PP4 |
| 41 | F | chrX:22.051.192-22.051.197 | c.70_74delGTCGT | p.(Val25Cysfs*24) | Exon 1 | Pathogenic PVS1+PM2+PP4 |

Abbreviation: Gender: F (Female); M (Male). Richards S, Aziz N, Bale S, Bick D, Das S, Gastier-Foster J, Grody WW, Hegde M, Lyon E, Spector E, Voelkerding K, Rehm HL; ACMG Laboratory Quality Assurance Committee. Standards and guidelines for the interpretation of sequence variants: a joint consensus recommendation of the American College of Medical Genetics and Genomics and the Association for Molecular Pathology. Genet Med. 2015 May;17(5):405-24.
